# The Biology of Nucleus-Forming Jumbo Phages

**DOI:** 10.1146/annurev-genet-111523-102019

**Published:** 2025-11

**Authors:** Erica A. Birkholz, Emily G. Armbruster, Joe Pogliano

**Affiliations:** Department of Molecular Biology, University of California, San Diego, La Jolla, California, USA

**Keywords:** phage nucleus, chimallivirus, early phage infection vesicle, EPI vesicle, phage tubulin, viral speciation, homing endonuclease, viral competition, viral factory, selfish gene, mobile genetic element

## Abstract

Nucleus-forming jumbo bacteriophages display a surprisingly intricate replication cycle inside of bacterial host cells, challenging the long-standing paradigm of prokaryotic simplicity. The phage nucleus encloses phage DNA in a protein shell, strictly uncouples transcription from translation, and facilitates selective protein import and messenger RNA (mRNA) export, serving the same major functions as the eukaryotic nucleus. Infection of host cells by these phages begins with the formation of a transcriptionally active membrane-bound early phage infection vesicle, demonstrating that these phages are capable of constructing subcellular compartments composed of lipids and proteins. Here, we review the current body of literature revealing the complexities of nucleus-forming phages and the history of the major discoveries. Studies of these phages are revealing new insights into basic principles of subcellular organization, viral speciation, and intracellular viral competition.

## INTRODUCTION

Subcellular organization was long thought to be a hallmark of eukaryotic cells and eukaryotic viruses, until new evidence began to reveal intricate organization within bacteria as well. However, viruses that infect bacteria, bacteriophages, were still thought to replicate very simply with no subcellular organization. This paradigm shifted with the discovery that certain bacteriophages also establish subcellular compartments in which their genome is fully enclosed by a proteinaceous shell composed of the phage-encoded protein chimallin (ChmA) (11, 22, 23, 65, 73, 92). This phage nucleus has many remarkable features that are similar to those of the eukaryotic nucleus. Replication and transcription of phage DNA occur inside the phage nucleus, mRNAs are exported to the cytoplasm where they are translated by host ribosomes, and proteins required for DNA-related processes are selectively imported into the phage nucleus. Another striking similarity to the replication cycle of eukaryotes is the phage tubulin–based spindle composed of PhuZ filaments, which are produced by nearly two-thirds of known nucleus-forming phages. This cytoskeletal structure is responsible for positioning the phage nucleus in the center of the host cell in *Pseudomonas* and *Erwinia* and, in many phages, rotating it. This is thought to facilitate capsid distribution around the phage nucleus surface and thereby more efficient DNA packaging. The initial step of infection by nucleus-forming phages begins with the formation of a transcriptionally active, membrane-bound compartment containing phage DNA known as the early phage infection (EPI) vesicle. These surprising discoveries demonstrate that the life cycle of these phages is remarkably complex and proceeds through the formation of two distinct subcellular compartments. In this review, we cover the discovery, diversity, and evolutionary implications of the novel subcellular structures of nucleus-forming phage biology.

## CHIMALLIVIRIDAE

Whole-genome phylogenetic trees indicate that nucleus-forming phages are a monophyletic group, which has been defined as the new viral family Chimalliviridae (92). Phylogenetic analysis of 66 phages encoding chimallin led to the identification of a set of genes that are highly conserved and define the core genome of chimalliviruses. The core genome consists of 68 genes that are largely encoded in 7 distinct blocks. The order of the blocks between different phages and the order of the genes within individual blocks are conserved. The phylogeny of individual core proteins suggests that these phages have not undergone extensive horizontal gene transfer, consistent with the physical segregation of the genome within the phage nucleus. Of these 68 genes, many encode generally conserved phage proteins, including structural proteins (capsids and tails) and large terminase, RNA polymerase (RNAP), and DNA polymerase genes. However, 21 of these genes are only found in Chimalliviridae phages, including the *chmA* gene itself. The functions of the vast majority of these genes are currently unknown, but they likely represent key proteins required for the phage nucleus replication pathway.

## JUMBO PHAGES AND PHAGE-ENCODED MULTISUBUNIT RNA POLYMERASES

Jumbo phages, phages with genomes >200 kb, were first described in 1963 (14, 34), long before the discovery of the phage nucleus in 2017 (23). Though large phages were originally thought to be rare due to their limited representation in phage collections, pioneering work using alternative phage isolation techniques designed to yield large phages (101) and later metagenomics (4) demonstrated that large phages are common in nature. Many jumbo phages encode two sets of rifampicin-resistant, multisubunit RNAPs (msRNAPs) (18, 24, 60, 66, 77, 79, 92, 106). Investigation into the roles of the two RNAPs in the phage life cycle revealed that one of the msRNAPs is a virion-packaged RNAP (vRNAP), which is injected into the host along with the phage DNA (113). This vRNAP transcribes the earliest genes of the infection, particularly *chmA* and the non-vRNAP (nvRNAP) that transcribes the middle and late genes (18). The nvRNAP of ΦKZ was studied in detail to determine the genes it transcribes and its composition of 5 subunits, including 4 with homology to the bacterial β/β′ subunits and a fifth with no known homologs outside of the phages encoding these msRNAPs (28, 90, 118). The structure of the nvRNAP of ΦKZ was resolved, revealing that the fifth subunit likely descended from an ancestral homolog of the σ factor based on its position within the protein complex, its structural homology, and its function (29).

## THE HISTORY OF THE DISCOVERY OF THE PHAGE NUCLEUS AND PHAGE SPINDLE

**Figure 1:**
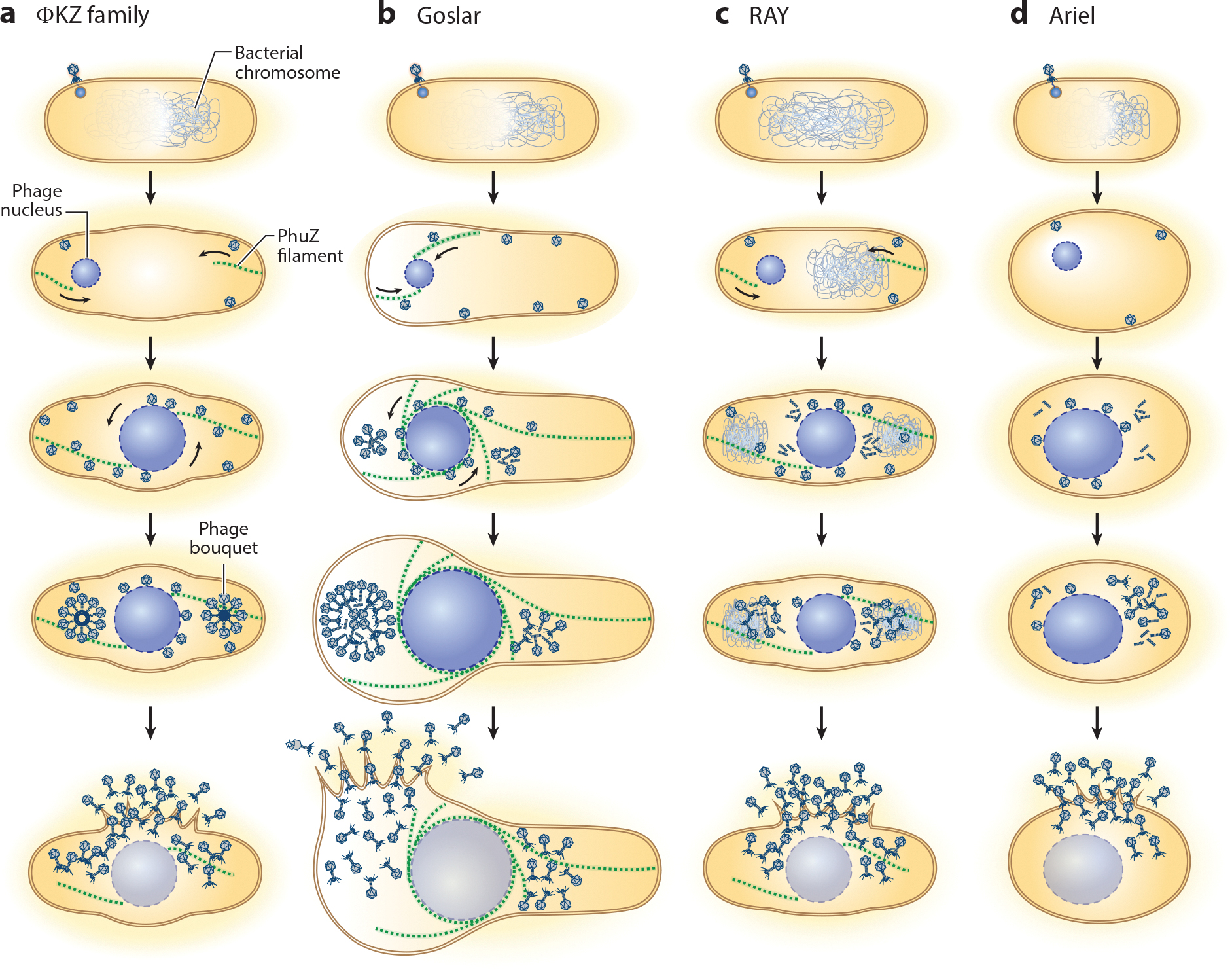
The diversity of chimallivirus replication cycle pathways. All viruses in this family establish a phage nucleus within which DNA replication occurs. (*a*) The *Pseudomonas* phage ΦKZ family, including ΦKZ, ΦPA3, and 201φ2–1. In the ΦKZ family, the bacterial chromosome is rapidly degraded and the cell swells in the middle. A tubulin spindle (PhuZ, *green filaments*) rotates and positions the phage nucleus at midcell. Capsids traffic along PhuZ filaments to the nucleus in *Pseudomonas* phages. Mature virions assemble into clusters called phage bouquets. Other chimalliviruses such as Goslar, RAY, and Ariel also form a nucleus but show distinct variations in the pathway. (*b*) *Escherichia coli* phage vB_EcoM_Goslar (Goslar) PhuZ filaments form a cytoskeletal vortex that surrounds and rotates the phage nucleus. (*c*) *Erwinia amylovora* phage RAY does not degrade the chromosome, and (*d*) *Vibrio parahaemolyticus* phage Ariel does not encode a PhuZ protein.

While jumbo phages were known to be interesting due to their unusual msRNAPs, the discovery of the PhuZ tubulin spindle constructed by phage 201φ2–1 in *Pseudomonas chlororaphis* provided the first cell biological evidence that they undergo a surprisingly complex replication cycle (58). PhuZ is a tubulin family found in many chimalliviruses (92) that has diverged from eukaryotic tubulins, bacterial FtsZ, and plasmid-encoded TubZ. PhuZ proteins from the ΦKZ family of viruses, including ΦKZ, ΦPA3, and 201φ2–1, have been intensively characterized (7, 22, 23, 43, 58, 119). In vitro, PhuZ assembles triple-stranded filaments (119) that display dynamic instability in a GTP-dependent manner (7, 43). Upon phage infection, PhuZ filaments form a bipolar spindle that extends from the poles of the *Pseudomonas* cell and positions replicating phage DNA in the center (22, 43, 58, 115). The PhuZ spindle also rotates the phage nucleus (23) and delivers capsids to its surface via treadmilling (21) (Figure 1a). Midcell positioning, phage nucleus rotation, and capsid trafficking are abolished with expression of the catalytic mutant of PhuZ. Despite all of these demonstrated functions, PhuZ is not a core gene, as it is absent from one-third of Chimalliviridae (92), and genetic studies indicate it is not essential for phage replication (49, 58). As an accessory gene, PhuZ likely plays a role in boosting phage reproduction efficiency.

## PHAGE NUCLEUS DISCOVERY

The phage nucleus was discovered through mass spectrometry of early abundant proteins produced during 201φ2–1 infection coupled with green fluorescent protein (GFP) tagging (23). One of the most abundant early proteins, now named ChmA, was tagged with GFP and observed to surround the phage DNA. The ChmA shell separates DNA processing proteins (e.g., DNA helicase, DNA ligase, RecA) encoded by both the phage and the host from the cytoplasmic host ribosomes and phage metabolic proteins such as thymidylate kinase and synthase. This protein shell uncouples transcription from translation and was therefore named the phage nucleus. Fluorescence in situ hybridization (FISH) with probes for host DNA and phage DNA demonstrated that the DNA in the phage nucleus is exclusively phage DNA (43). The phage nucleus also serves as a hub for packaging genomes into capsids that are delivered by PhuZ filaments as the nucleus rotates (21). Cryo-focused ion beam milling coupled with cryo-electron tomography (cryoFIB-ET) showed the phage nucleus to be an enclosed compartment surrounded by a regular chain of 6-nm proteins and revealed partially assembled capsids on the bacterial inner membrane as well as fully assembled virions in a group adjacent to the phage nucleus (23, 64). CryoFIB-ET also provided a high-resolution view of capsids containing varying amounts of DNA distributed around the periphery of the phage nucleus. ΦKZ and ΦPA3 were subsequently shown to replicate by forming a phage nucleus and spindle (22, 87). ΦKZ infection progression was also analyzed, using transmission electron microscopy on fixed embedded cells, where they observed the phage nucleus similarly in the center of the cell (26).

## THE STRUCTURE OF THE CHIMALLIN SHELL AND IMPLICATIONS FOR FUNCTIONS

**Figure 2:**
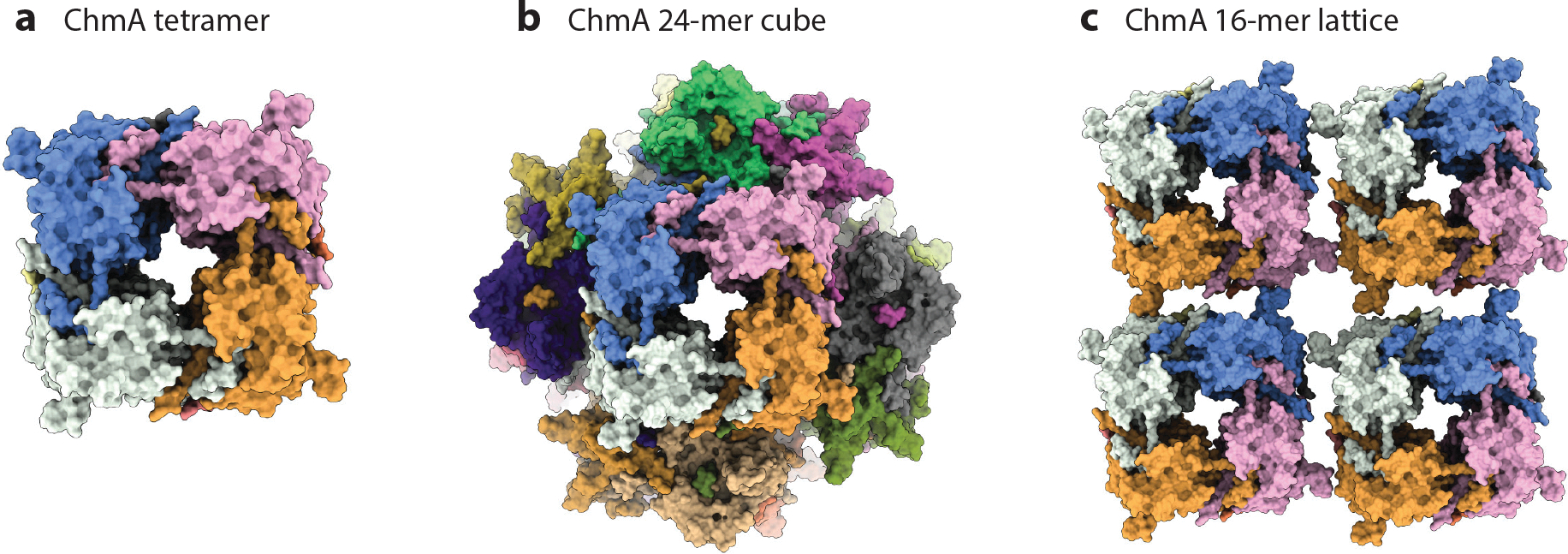
Chimallin (ChmA) protein structures and higher organization. (*a*) Homotetramer of 4 Goslar ChmA proteins. (*b*) Cube assembly of 24 Goslar ChmA proteins as observed in vitro. (*c*) Lattice structure of 16 Goslar ChmA proteins, which is repeated to form the surface of the phage nucleus (65).

The structure of the protein shell segregating phage DNA appears to be highly conserved. CryoFIB-ET of phage nuclei in situ and single particle analysis of purified protein in vitro revealed that the phage nucleus is primarily composed of ChmA, which forms homotetramers that are assembled into a square lattice (65, 71, 89) (Figure 2). The ChmA N-terminal segment interacts with other monomers in the same tetramer, while the C-terminal segment interacts with neighboring tetramers. Deletion of any of these segments ablates the self-assembly of ChmA shells in vitro, which supports their role in linking the lattice of tetramers together (65). The C-terminal interactions are similar to interactions between the toxin–antitoxin pair AtaT and AtaR, suggesting that ChmA may have evolved from a toxin–antitoxin system.

## VIRION ASSEMBLY AND GENOME PACKAGING

The newly assembled capsids of these nucleus-forming jumbo phages must reach the shell of the phage nucleus to receive a genome, but they are assembled on the bacterial inner membrane (23). Studies with *Pseudomonas* chimalliviruses demonstrated that capsids are trafficked to the phage nucleus by the PhuZ spindle (21). Fluorescent capsids were visualized in a line along the PhuZ filaments, while expression of mutant PhuZ resulted in a large number of empty capsids clustered around the spindle distal to the phage nucleus (21). Functional PhuZ filaments rotate the nucleus while distributing capsids on its surface for genome packaging. Then the capsids detach from the phage nucleus and form spherical arrangements of maturing virions termed phage bouquets, with the capsids on the outside of the sphere and the tails pointing inward (20). Over the course of the infection, the amount of DNA in the bouquets increases as the concentration of DNA inside the phage nucleus decreases, indicating that the viral genomes are being transferred into the maturing virions. GFP-tagged tail proteins localize inside the bouquets, demonstrating the orientation of the virions. The exclusion of cytoplasmic proteins from the bouquets suggests that the structure is very densely packed. A large bouquet with tails attached to capsids as well as a small bouquet that did not yet contain tails was visualized using cryoFIB-ET (20). Infection in the presence of a dominant negative PhuZ catalytic mutant showed that bouquets retain their position in relation to the phage nucleus, indicating that they do not depend on the PhuZ spindle for positioning. However, the PhuZ mutation causes the filament to become static, so capsids are not efficiently trafficked to the phage nucleus, reducing the size and number of bouquets observed (20).

## PHAGE NUCLEUS DIVERSITY

Although the structure of the phage nucleus shell is highly conserved, there is considerable diversity in the chimallivirus replication pathway. Phage vB_EcoM_Goslar, hereafter referred to as Goslar, is a chimallivirus that infects *Escherichia coli* (57). Goslar exhibits the hallmarks of a chimallivirus replication cycle with both a nucleus-like compartment and a tubulin-based cytoskeleton that rotates it (11). A distinct feature of Goslar replication that highlights the diversity of the chimallivirus replication cycle is the organization of PhuZ filaments into a vortex-like array rather than a bipolar spindle (Figure 1b). This cytoskeletal structure wraps around the entire phage nucleus and projects radially toward the membrane in all directions. Rotation of the phage nucleus is inhibited by expression of a catalytically defective PhuZ protein, demonstrating that dynamic PhuZ filaments are necessary to generate the rotation. Live-cell time-lapse fluorescence microscopy revealed that the direction of phage nucleus rotation is determined by the generation of force through these filaments pushing against the membrane. Slight inequality in the timing and strength of force delivered by developing filaments begins a self-reinforcing organization of the observed cytoskeletal vortex. In vitro, purified eukaryotic microtubules in the presence of *Chlamydomonas* dynein-c (109) and endogenous microtubules in spatially confined droplets of *Xenopus* oocyte extract (110) spontaneously organize into a vortex array, supporting the hypothesis that this characteristic of microtubule organization is conserved across more than one kingdom of life. Factors that control the organization of PhuZ filaments into a bipolar spindle or a cytoskeletal vortex by different phages are currently unknown.

Another characteristic of Goslar infection that is distinct from the *Pseudomonas* chimalliviruses is the positioning of the phage nucleus within the host cell. The *Pseudomonas* phages center the nucleus via the bipolar spindle, while the Goslar phage nucleus can be found anywhere along the lateral axis of the cell (11). Expression of a catalytically defective PhuZ protein does not affect nucleus positioning, suggesting that the Goslar PhuZ vortex array serves the function of nuclear rotation but not positioning. Goslar forms phage bouquets that are similar to those observed in the three *Pseudomonas* chimalliviruses; however, the bouquets in Goslar are much larger and contain capsids in the center of the bouquets rather than only on the periphery. This suggests that Goslar bouquets accommodate internal capsids that are inversely oriented relative to the outer layer (Figure 1b).

The *Erwinia* phage RAY is a distant relative of Goslar and the ΦKZ-related chimalliviruses (92). Fluorescence microscopy and cryoFIB-ET demonstrated that many of the chimallivirus replication mechanisms are conserved in RAY, including the formation of a 6-nm thick shell of chimallin that surrounds phage DNA and separates transcription and translation. A characterization of 10 proteins that had not previously been studied in chimalliviruses revealed several differences from the *E. coli* and *Pseudomonas* chimalliviruses. First, the host chromosome is not degraded and is excluded from the phage nucleus. This has only been observed previously with the *Serratia* phage PCH45 (73) and suggests that the host chromosome is not a barrier to building a phage nucleus. Second, both the PhuZ spindle of RAY and the host chromosomes likely impact phage nucleus positioning. Third, RAY PhuZ assembles 5-stranded tubes with a central lumen, which has never before been observed for any phage tubulin. Fourth, unlike the *Pseudomonas* and *E. coli* chimallivirus nuclei, which are consistently rotated by the PhuZ spindle, rotation of the RAY nucleus was rarely observed. Finally, maturing RAY viral particles do not assemble into bouquets (Figure 1c).

The *Vibrio* phage Ariel and the *Erwinia* phage Asesino do not encode a PhuZ protein yet still replicate by forming a phage nucleus (93, 112). During Ariel infection, the host genome is degraded, and the host cell becomes spherical. The phage nucleus occupies the center of the small spherical cell, possibly as an incidental effect of the ribosomes translating mRNA as it is exported from the nuclear shell, resulting in a spatial buffer between the nuclear shell and the cell membrane (Figure 1d). By contrast, Asesino does not degrade the host genomes, which appear to contribute to Asesino positioning within the cell (93).

## PHAGE NUCLEUS IMPORT AND EXPORT

The phage nucleus must export mRNA and import proteins as well as nucleotides to support DNA and RNA synthesis, so it must have channels to facilitate transport across the ChmA lattice. To date, although several phage proteins involved in protein import and one facilitating mRNA export have been identified (41, 55, 82), no distinct channels in the phage nucleus have been observed in cryoFIB-ET. Instead, the ChmA lattice of phages Goslar, ΦPA3, and 201φ2–1 has small pores in the center of the homotetramers with an average reported diameter of 1.4 nm for the phage 201φ2–1 (65, 71, 89). These pores are too small to facilitate protein trafficking but could allow the transport of mRNA as well as small molecules such as nucleotides. The potential for transcripts to be transported through the central pores is supported by their negative charge (65, 89). Elucidating the mechanism of molecular transport across the ChmA lattice, including potential roles for these pores, is a key area of future exploration. There are many remaining questions to investigate regarding the assembly and function of ChmA and how it relates to the operation of the phage nucleus, including (*a*) what are the requirements for initial phage nucleus assembly in vivo, (*b*) how does the selective protein import machinery interact with the ChmA shell, and (*c*) how do capsids dock to the phage nucleus surface for genome packaging? Answering these questions will require discovering all of the proteins that interact with the phage nucleus shell.

A useful tool for probing the selective import dynamics of the phage nucleus was stumbled upon in 2021 (87). One type of GFP in particular, GFPmut1, but not other GFP variants, was found to be naturally imported into the phage nucleus of ΦKZ but excluded from the nuclei of ΦPA3 and 201φ2–1. Amino acid substitutions revealed that altering two surface residues, M153 and F99, changed the import of the fluorescent protein into the ΦKZ nucleus. To demonstrate whether GFPmut1 could be used as a tool to import cytoplasmic proteins into the ΦKZ nucleus for further investigations, GFPmut1 was fused to mCherry and a DNA nuclease. This resulted in the import of both proteins in a functional state (87).

Utilization of the differential GFP import discovery led to the characterization of protein importer of chimalliviruses A (PicA), which is an essential chimallivirus protein required for the selective import of proteins into the nucleus (82). By fusing GFPmut1 to a cytoplasmic toxic nuclease, ΦKZ mutants were isolated with altered protein import that did not allow GFPmut1 into the nucleus, protecting the viral genomes from the nuclease. ΦKZ protein import mutants identified with this method had amino acid substitutions in an uncharacterized protein encoded by a core Chimalliviridae gene only found in nucleus-forming phages. This protein was named PicA and found via CRISPR interference through antisense RNA targeting (CRISPRi-ART) to be essential for phage replication in *E. coli* chimallivirus Goslar (82). Mutations on the surface of PicA altered the selectivity and rate of protein import into the phage nucleus. The characterization of this import system demonstrated that cargo proteins are targeted to the phage nucleus not through a terminal signal sequence, as in many other protein trafficking systems, but through amino acids on the surface of the folded protein. The amino acids that determine targeting differ between chimallivirus species (82). Fluorescently tagged PicA forms a single punctum on the phage nuclear shell, where it also colocalizes with a stalled transport-defective cargo protein, indicating that PicA interacts with protein import substrates and that protein import likely occurs at a single location on the nuclear shell. PicA was also identified using a different genetic selection and referred to as Imp1, and additional phage proteins were implicated in nuclear protein import (55), but understanding the full mechanistic details of protein import remains an exciting direction for future research.

Several new approaches have been successfully deployed to identify the phage-encoded factors that interact with the phage nucleus and participate in various steps of the chimallivirus replication pathway or interactions with host cells. Size exclusion chromatography and mass spectrometry (SEC-MS) were used to create a phage protein and host protein interaction network, which found phage proteins that likely interact with ΦKZ proteins, including chimallin and inner body proteins, or interact with host proteins (44). Grad-seq is another exciting approach to finding phage proteins that form large complexes inside of the cell (47, 105). When applied to ΦKZ, Grad-seq found several proteins that interact with host ribosomes. One of these proteins, ΦKZ014, binds tightly to ribosomes, where it is likely involved in the initial steps of taking over the host cell (45). Proximity labeling with biotin identification (BioID) utilizes the fusion of a promiscuous biotin ligase to a protein of interest, allowing the detection of weak or transient interactions (98). Potential ChmA interaction partners were identified using ChmA fused to miniTurboID. Six such proteins were confirmed to localize to the phage nucleus shell (42). One of these proteins, ChmB, was shown to interact directly with ChmA and several other proteins in biochemical pulldown experiments. Although the function of ChmB is currently unknown, it appears to play a role in proper phage nucleus growth and DNA replication. Additional key phage nucleus proteins identified in this study include PicA (55, 82), ChmC (41, 55), and Imp3 (55). ChmC is an RNA-binding protein and may be involved in exporting RNA from the nucleus (41), while PicA and Imp3 are involved in nuclear protein import (55, 82). Another innovative approach is the use of antisense oligonucleotides (ASOs) to specifically knock down protein expression during phage infection (46). ASOs were recently used in combination with RNA-seq and fluorescence microscopy to identify many ΦKZ proteins essential for replication, revealing RNase H as a key factor required for replication.

## EVADING HOST DEFENSES

The phage nucleus provides protection from host immune defenses (73, 78, 87). ΦKZ and ΦPA3 can evade restriction modification (RM) enzymes (types I and II) as well as CRISPR-Cas systems, including the types found in *Pseudomonas* (types I-C and I-F) and those not naturally occurring in the host (types II-A and V-A) (78). In vitro, RM enzymes and Cas9 were able to cut ΦKZ DNA, but, in vivo, the components of those host immune systems were excluded from the phage nucleus. Fusion of phage-encoded RecA, which is naturally imported into the phage nucleus, to the immunity enzymes EcoRI and Cas9 resulted in successful reduction of phage titer by EcoRI, but Cas9 accumulated at the periphery of the phage nucleus and did not produce a knockdown of phage replication (78). While the phage nucleus provides physical protection from DNA-targeting host defenses, the RNA-targeting Cas13 is able to cleave transcripts exported from the phage nucleus for translation in the cytoplasm and thus severely inhibit ΦKZ replication. *Serratia* chimallivirus PCH45 was also found to evade DNA-targeting type I CRISPR systems while being susceptible to an RNA-targeting type III system (73). Bioinformatic analyses uncovered a significant enrichment of chimallivirus-targeting spacers in type III compared to type I systems, consistent with the phage genome being sequestered in the cytoplasm by the phage nucleus and therefore inaccessible to DNA-targeting type I CRISPR systems (73).

## EARLY PHAGE INFECTION VESICLES

**Figure 3:**
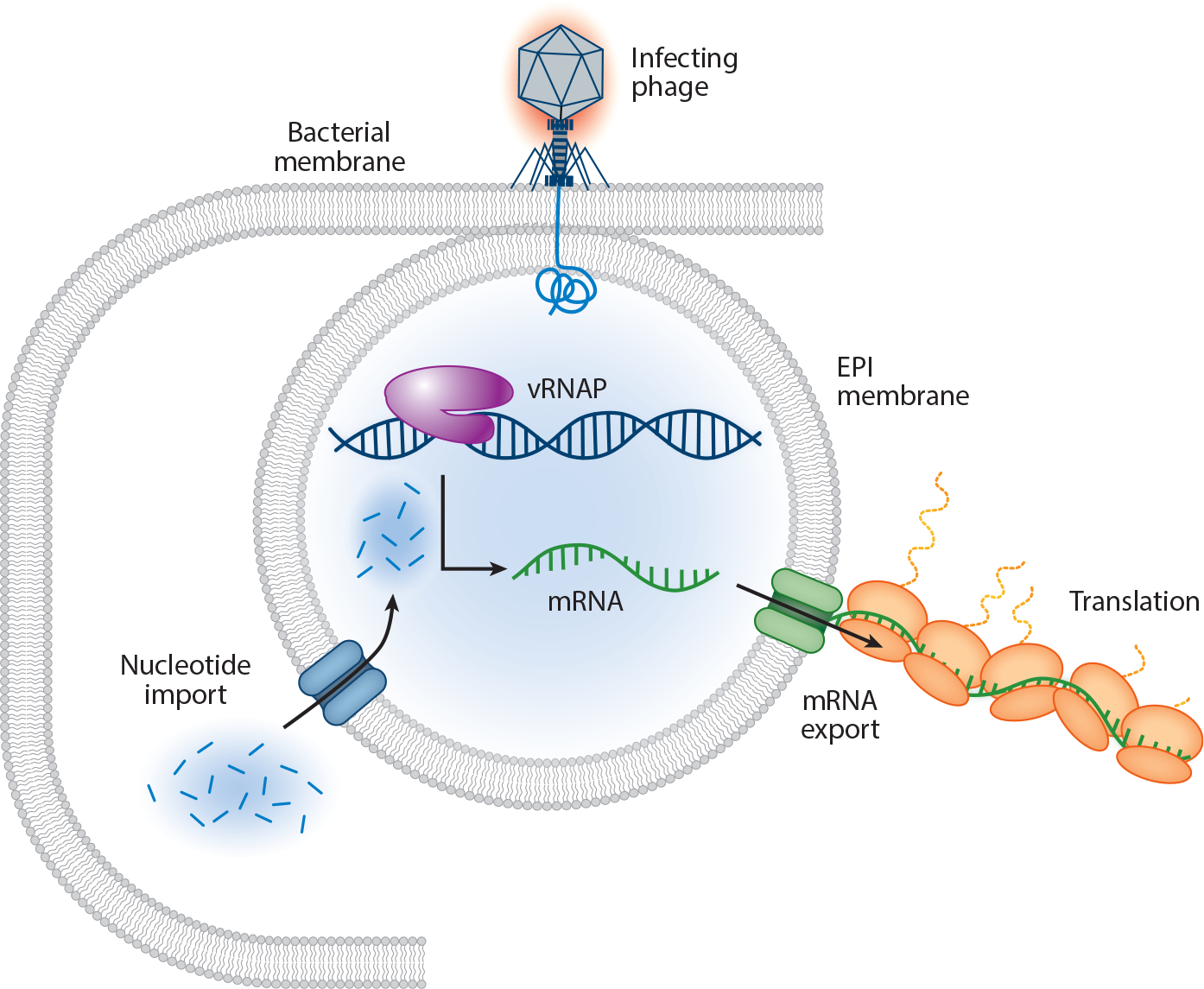
The EPI vesicle was first discovered in the *Escherichia coli* phage Goslar, where a membrane-bound compartment was revealed upon inhibition of the expression of ChmA. The EPI is transcriptionally active, indicating that it must have mechanisms for importing nucleotides and exporting mRNA. Abbreviations: ChmA, chimallin; EPI, early phage infection; Goslar, vB_EcoM_Goslar; mRNA, messenger RNA; vRNAP, virion-packaged RNAP.

The phage nucleus shields viral genomes from DNA-targeting host defenses such as restriction enzymes and CRISPR-Cas systems in the cytoplasm (73, 78, 87). Yet, how the phage genome is protected from such defenses before phage nucleus assembly remained unknown for several years, due in part to the lack of genetic tools compatible with chimalliviruses. Studies using fluorescence microscopy, cryoFIB-ET, and RNA-seq in combination with the powerful new gene expression knockdown technology (2) CRISPRi-ART have revealed that the injected genome of *E. coli* chimallivirus Goslar is initially enclosed in a membrane-bound, transcriptionally active organelle, the EPI vesicle (6). These vesicles contain injected virion proteins as well as the viral DNA, including the vRNAP. The vRNAP drives expression of early phage transcripts from within the vesicle, which then must be exported to the cytoplasm for translation by host ribosomes. Thus, there must be as-yet-unidentified channels for nucleotide import and transcript export. Similar to a eukaryotic nucleus, the membrane-based EPI vesicle is transcriptionally active and allows the two-way exchange of molecules (Figure 3).

The early genes expressed from the EPI vesicle encode all the proteins needed for phage nucleus assembly, including ChmA itself, PicA (which regulates protein import into the nucleus) (82), and ChmC (which regulates mRNA export to the cytoplasm) (41). The genome within the EPI vesicle is transferred into the nascent phage nucleus, which initially assembles immediately adjacent to the vesicle. Thus, the phage genome is completely shielded at all stages of infection, explaining its ability to bypass DNA-targeting (Cas9) host defense systems. If the phage nucleus cannot assemble, the genome remains within the EPI vesicle and cannot (*a*) express the viral late-infection genes such as the major capsid protein or (*b*) replicate the viral genomes. Thus, the phage nucleus is intrinsically essential for phage replication, in addition to its role in protecting the viral genomes from host defenses.

Notably, EPI vesicles had been previously observed in cells infected by diverse chimalliviruses, but they were completely mysterious, with nothing known about their composition, origin, or function (26, 65, 96). Now, with the EPI vesicle established as a phage-generated, transcriptionally active organelle in the early life cycle of the *E. coli* phage Goslar, it is clear that the EPI vesicle is a conserved stage across the Chimalliviridae family. EPI vesicles were also confirmed to form during ΦKZ infection (5, 83).

The discovery of the EPI vesicle opens a new field in prokaryotic biology. It is the first known example in prokaryotes of a genome fully enclosed in a membrane-bound compartment that separates transcription from translation. It is also the first example of a membranous organelle produced by a phage. Many eukaryotic viruses hijack host membranes to generate viral compartments that facilitate transcription and protect the genome from host defenses (54, 100, 103). The discovery of the EPI vesicle demonstrates that this strategy is universal for viruses across host kingdoms. Many questions are raised by the discovery and initial characterization of the EPI vesicle: What is the mechanism of vesicle formation? How are nucleotides imported and mRNA exported? How does the genome transfer from the EPI vesicle to the newly assembled phage nucleus? Does the EPI vesicle have a function in the life cycle after the genome is transferred? These questions are an exciting new path for investigation in the study of Chimalliviridae biology.

## VIRAL SPECIATION FACTORS

The discovery of the intricate organization of chimalliviruses raised the question of how they would interact during a coinfection of the same host cell. Experimental and ecological observations reveal that viral coinfections are abundant in nature (27, 30–32, 36, 72, 104), so investigating the intracellular interactions of viruses is vital to understanding viral speciation and evolution. Once a coinfection is successfully established, viruses may recombine to produce hybrids with increased or decreased virulence, as demonstrated for influenza (35, 68, 69, 107, 116). Genetic exchange between bacteriophages was previously thought to be unrestricted (17, 51, 75). If that were the case, we would expect phage genomes to cluster into homogeneous gene pools separated strictly by host tropism, and while that is a significant determinant of phylogeny, phage evolution is more complex (13, 30). The intracellular separation of viral factories has been implicated in reproductive isolation that may support speciation between vaccinia viruses (15, 53, 70, 91) and between herpes simplex virus 1 (HSV-1) viruses (114), but no universally applicable speciation factors were proposed. Virus phylogeny is currently dependent on comparative sequence analyses (3, 16, 50, 59, 67), but the idea of defining viral species using the Biological Species Concept (33, 76) has also been proposed (13). This concept says that organisms belong to different species when they belong to separate gene pools. Two organisms are distinct species when their mating, or recombination of genetic traits, is no longer possible or productive. As two organisms diverge genetically due to the limitation of genetic recombination caused by a reproductive isolating mechanism, genetic variations that are incompatible with each other, known as Dobzhansky–Muller genetic incompatibilities, can accumulate, which further isolate the gene pools and drive speciation (33, 84–86).

Geographical separation and host tropism are known to be strong reproductive isolating mechanisms for viruses (36, 81, 99) since coinfection is required for genetic recombination (95, 117). However, even when two viruses share the same environment and are able to infect the same host, they may not achieve coinfection due to superinfection exclusion, which prevents productive infection after an initial infection has already occurred (1, 37), thereby separating gene pools. Still, a key question remained: How do viruses that successfully infect the same cell speciate?

Studies of chimallivirus intracellular competition led to the discovery of two general principles of viral speciation that can be applied to any virus or intracellular parasite (19). Subcellular genetic isolation occurs when spatial barriers, such as the phage nucleus, limit genetic exchange between coinfecting viruses. When two chimalliviruses infect the same cell, two independent nuclei are established, and they remain separated throughout the lytic cycle (19). This holds true for distantly related chimalliviruses and for identical ones that independently inject their DNA into the same host cell. Since we now know that the phage DNA first enters the cell via a membrane-bound EPI vesicle (5, 6, 83) and is then transferred to the phage nucleus, subcellular genetic isolation is maintained throughout the chimallivirus life cycle. Virogenesis incompatibility occurs when divergent proteins from coinfecting viruses interfere with the production of viable progeny (19). The first demonstration of this was shown for PhuZ tubulins encoded by ΦKZ and ΦPA3. During coinfection, these divergent tubulins coassemble into nondynamic filaments that do not properly position or rotate the phage nucleus, which is predicted to result in less efficient reproduction. Both of these mechanisms, subcellular genetic isolation and virogenesis incompatibility, create barriers to genetic exchange that lead to divergence and the speciation of viruses, representing universal speciation factors that are applicable to viruses infecting all domains of life (19).

## INTRACELLULAR PHAGE COMPETITION: HOMING ENDONUCLEASES AS WEAPONS

The subcellular genetic isolation of chimallivirus genomes during replication makes for an interesting study of the interactions of mobile genetic elements carried by these phages. Phages commonly contain self-splicing introns (61, 108) encoding homing endonucleases that mobilize the intron to invade the unoccupied locus in a related genome, the intron^−^ allele. Homing endonucleases target the DNA at their homing site, causing a DNA break in the intron^−^ allele, triggering recombination, which results in unilateral gene conversion with the loss of the homing site (39, 40, 108). While homing endonucleases can be freestanding, in phages, they are often found within group I introns (8, 9, 38, 108). Those self-splicing elements allow the homing endonuclease to invade highly conserved coding sequences by ensuring that the essential genes of the host remain functional (40, 108). It is advantageous for the introns to invade essential genes at conserved residues, such as enzyme active sites, so that any imperfect attempt to delete the intron would lead to a nonfunctional protein (38, 39, 88).

The most common mobile introns in phages interrupt genes involved in DNA metabolism (39). In every domain of life, group I introns interrupt the genes that encode mRNA, ribosomal RNA (rRNA), and transfer RNA (tRNA) (40, 52, 88). When these introns invade a related genome by creating a double-strand break (10, 25, 97, 111) or a single-strand nick (48, 56, 62, 64, 80, 102), homologous recombination leads to repair, which is less efficient between more divergent genomes (74, 94, 111).

Phage-encoded group I introns containing a homing endonuclease have been studied in the context of coinfecting phages. In the case of the *Bacillus* phage SPO1, the presence of a homing endonuclease has been shown to promote recombination and the transfer of neighboring genes to a related phage during coinfections in a process called marker exclusion (48, 63), although there was no evidence that this provided any fitness advantage to the phage encoding the intron.

**Figure 4:**
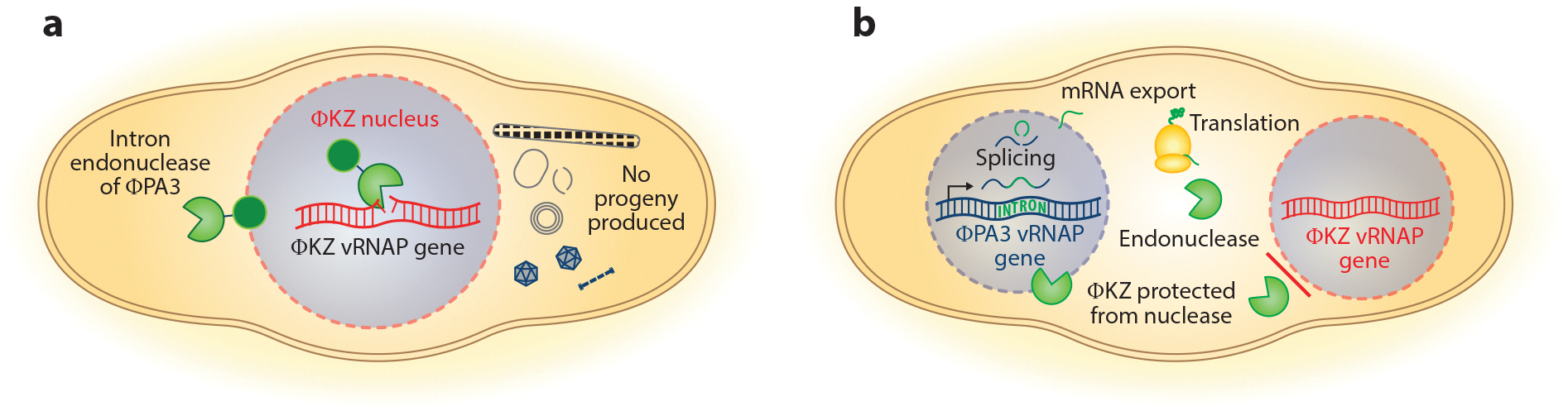
Weaponized intron endonuclease action and divergence of selective import. (*a*) Artificial import of the intron endonuclease encoded by ΦPA3 (*green Pac-Man*) into the ΦKZ nucleus using GFPmut1 (*green circle*) results in nicking of the vRNAP gene and inhibition of capsid assembly. Aberrant capsid protein assemblies are represented by the gray objects. (*b*) The differences in selective nuclear protein import between closely related coinfecting phages ΦPA3 (*blue phage nucleus*) and ΦKZ (*red phage nucleus*) result in the exclusion of the ΦPA3 nuclease, which is harmful to ΦKZ, allowing them to coexist as distinct species. Abbreviations: mRNA, messenger RNA; vRNAP, virion-packaged RNAP.

These mobile intron systems have been known as selfish mobile elements, but the first report of a selective advantage for an intron^+^ virus over an intron^−^ virus was recently published, calling into question the idea of selfish genes (12). Using the model chimalliviruses ΦKZ and ΦPA3 that coinfect *Pseudomonas aeruginosa*, a homing endonuclease encoded within an intron interrupting a ΦPA3 RNAP subunit gene was found to provide a competitive advantage to ΦPA3 through interference competition (12). This homing endonuclease is normally excluded from the ΦKZ nucleus, but when it is artificially imported by fusing it to GFPmut1 it is able to target the intron^−^ vRNAP gene in ΦKZ, interfering with capsid assembly by an unknown mechanism (Figure 4a). Compared to ΦPA3 with a mutated homing endonuclease, ΦPA3 that encodes a functional copy of the homing endonuclease can better defend its niche from ΦKZ invasion by decreasing the number of ΦKZ virions produced. This was the first demonstration of a competitive advantage conferred by a mobile intron to the host virus, showing that mobile genetic elements previously believed to be wholly selfish are actually capable of being advantageous to their host organism.

We hypothesize that the mechanisms of selective protein import are likely shaped by the evolutionary pressure to exclude enzymes harmful to the phage DNA (12, 82). The discovery of weaponized homing endonucleases predicts that ΦPA3 and ΦKZ would evolve different specificities for protein import to avoid importing toxic endonucleases native to the other phages (Figure 4b). In support of this hypothesis, we know of many proteins that are differentially imported between these two phages, including the ΦPA3 endonuclease, which can target the ΦKZ genome (12, 19, 82, 87). Though why these two related phages would display such disparate protein import selectivity was initially puzzling, this homing endonuclease model provides an explanation and predicts that such disparities must evolve for both phages to coexist.

## CONCLUSION

From the time of their discovery, members of the Chimalliviridae family have been remarkable examples of unprecedented sophistication among bacteriophages. When large genome phages were initially isolated, they were believed to be extremely rare, but further discoveries have revealed that they are prevalent in diverse ecosystems (4, 101). While most smaller genome phages rely on host transcription machinery, chimalliviruses encode two msRNAPs (18, 24, 60, 66, 77, 79, 92, 106). For nearly a century, phages were believed to replicate in the bacterial cytoplasm without much organization, but chimallivirus discoveries have completely reshaped the understanding of phage biology. Chimalliviridae phages make two transcriptionally active organelles that separate transcription from translation: the membrane-bound EPI vesicle and the proteinaceous phage nucleus. These phages have also evolved a unique mechanism for selective protein import across the chimallin protein lattice while strictly excluding host defense proteins to protect the phage DNA. The majority of chimalliviruses even encode their own PhuZ tubulin, which dynamically positions the nucleus and facilitates capsid packaging. New techniques such as cryoFIB-ET, Grad-seq, SEC-MS, BioID, CRISPRi-ART, and ASOs are facilitating new insights into chimallivirus replication and evolution. Chimalliviruses are also proving to be useful for understanding general principles of viral speciation and evolution. Future research on the Chimalliviridae family will likely continue to uncover unexpected complexity and novel mechanisms underlying phage life cycles.
